# Epidemiology and Clinical Profile of Brachial Plexus Injuries in Semi-Urban Punjab: Insights From a Retrospective Study at a Tertiary Care Center

**DOI:** 10.7759/cureus.105691

**Published:** 2026-03-23

**Authors:** Mohd Altaf Mir, Rahul Kaushik, Devinder Kumar, Labhanshi Aggarwal, Taruna Singh, Raghesh Rajkumar, Lohith Y N, Ankit Negi, Salot Jainam Alpesh Kumar

**Affiliations:** 1 Burns and Plastic Surgery, All India Institute of Medical Sciences, Bathinda, Bathinda, IND

**Keywords:** brachial plexus injury, clinical profile, epidemiology, peripheral nerve injury, punjab, road traffic accidents

## Abstract

Background: Adult traumatic brachial plexus injury (BPI) is a severe peripheral nerve injury that frequently results in significant upper limb disability in young individuals. The epidemiology varies with different geographical locations. This study aimed to analyze the demographic profile, mechanisms of injury, injury severity patterns, referral timelines, and reconstructive burden of adult traumatic BPI in a tertiary care institution of northern India.

Methods: A retrospective observational study was conducted at the All India Institute of Medical Sciences (AIIMS), Bathinda, Punjab, India, over a two-year period. Adult patients (≥18 years) with clinically and radiologically confirmed traumatic BPI were included. Cases of obstetric and iatrogenic brachial plexus injury were excluded. Data regarding age, sex, mechanism of injury, level and severity of plexus involvement, time to presentation, associated injuries, and management modality were collected. Descriptive statistical analysis was performed.

Results: Seventy-five adult patients were included, with a mean age of 29.1 years; 69 (92%) were male. Road traffic accidents were the predominant cause, with two-wheeler accidents accounting for 78.7% of cases. Complete C5-T1 involvement was observed in 59 patients (78.7%). Forty-five patients (60%) presented more than six months after injury. At the time of analysis, 51 patients (68%) had undergone surgical intervention, including primary nerve transfer and secondary reconstructive procedures, while 24 (32%) had not undergone operative treatment.

Conclusion: Adult traumatic BPI in this semi-urban North Indian cohort was predominantly associated with high-energy two-wheeler accidents and a high proportion of complete plexus injuries, with frequent delayed presentation. These findings highlight the substantial reconstructive demand in tertiary centers and underscore the need for improved referral pathways and trauma prevention strategies.

## Introduction

Adult traumatic brachial plexus injury (BPI) is one of the most severe peripheral nerve injuries, frequently resulting in profound upper limb dysfunction and long-term disability. These injuries predominantly affect young, economically productive individuals and impose substantial functional and socioeconomic burdens if timely intervention is not provided [1]. Despite advances in microsurgical techniques and nerve transfer strategies, functional recovery remains highly dependent on injury severity and timing of reconstruction.

High-energy trauma, particularly road traffic accidents (RTAs), is the leading cause of adult BPI worldwide. Multiple epidemiological studies have consistently demonstrated a marked male predominance and a strong association with motorcycle-related accidents [2-6]. In an Indian cohort, Jain et al. reported that more than 80% of traumatic BPI cases were attributable to RTAs [2]. Similar trends have been observed in large international series, including the multicenter Chinese study by Li et al. [7] and the systematic review by Kaiser et al. [5], both of which identified high-velocity vehicular trauma as the principal etiology of severe adult brachial plexus injuries requiring surgical repair.

The severity and anatomical pattern of injury are closely related to the mechanism and magnitude of trauma. High-velocity traction forces, particularly in motorcycle crashes, can produce multi-root involvement or complete C5-T1 palsy due to violent shoulder depression combined with contralateral cervical displacement [5,7]. National-level epidemiological data from England and Wales have demonstrated the persistent burden of traumatic BPI over several decades, with a significant proportion of patients sustaining severe injuries requiring operative intervention [6]. These findings highlight the continued public health importance of traumatic BPI even in developed trauma systems.

Although surgical reconstruction has evolved considerably, delayed presentation remains a major challenge, particularly in low- and middle-income countries. Early intervention within the first 3-6 months following injury is widely considered critical for optimal reinnervation and functional recovery [1]. However, delayed referral to tertiary centers often results in advanced muscle denervation, limited reconstructive options, and potentially inferior outcomes [2,5]. Understanding regional referral patterns is therefore essential for improving patient pathways and optimizing surgical planning.

While several epidemiological studies have described traumatic BPI in urban or national populations, region-specific data from northern India, particularly semi-urban regions such as Punjab, remain limited. Variations in traffic patterns, two-wheeler density, healthcare accessibility, and health-seeking behavior may influence both injury severity and timing of presentation.

Accordingly, this study aimed to analyze the epidemiological profile, injury severity distribution, referral patterns, and reconstructive workload of adult traumatic brachial plexus injuries presenting to a tertiary care center in the Malwa region of Punjab between January 2023 and March 2025.

## Materials and methods

Study design and setting

This retrospective observational study was conducted in the Department of Burns and Plastic Surgery at the All-India Institute of Medical Sciences (AIIMS), Bathinda, Punjab, India. This study evaluated adult patients with traumatic brachial plexus injuries who presented between January 2023 and March 2025. This study aimed to analyze demographic characteristics, mechanisms of injury, injury severity patterns, referral intervals, and reconstructive burden among patients from the Malwa region of Punjab.

Study population and sample size

All consecutive adult patients aged ≥18 years who were clinically and radiologically diagnosed with traumatic brachial plexus injury during the study period were included. A consecutive non-probability (census) sampling approach was adopted, and all eligible cases presenting within the defined time frame were screened for inclusion. Formal sample size estimation was not performed.

**Figure 1:**
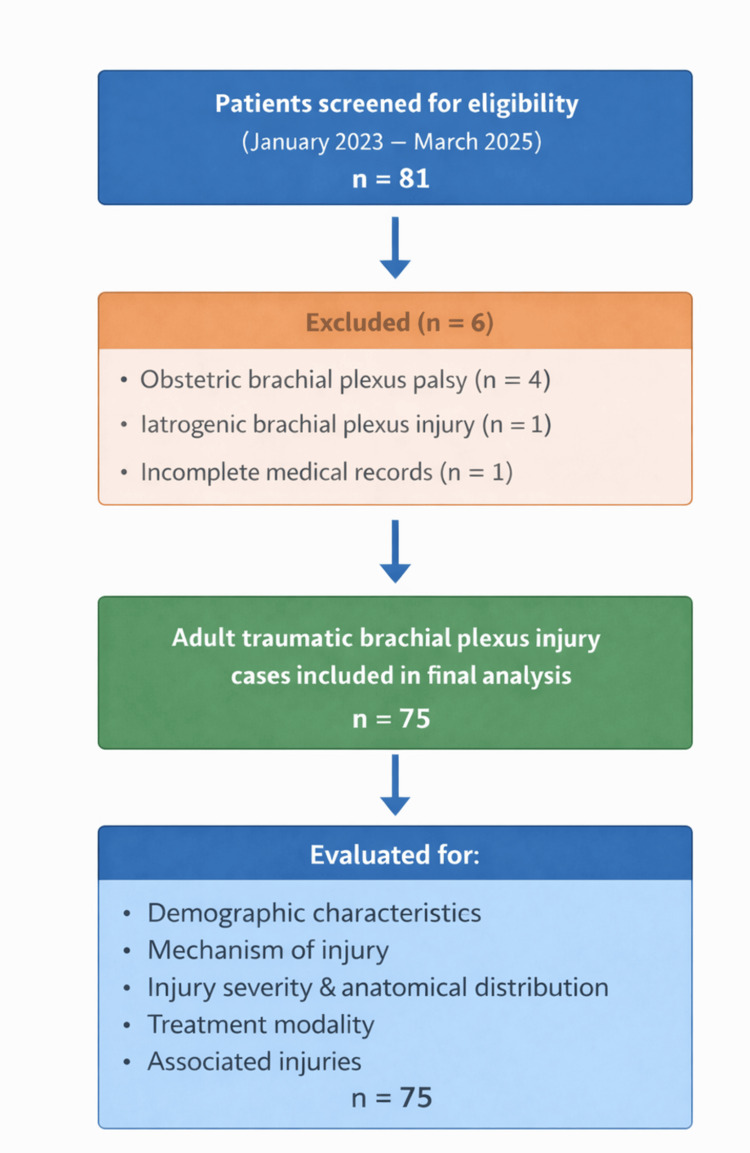
Flow diagram illustrating the patient recruitment and selection process for the study

Patients with obstetric brachial plexus palsy, iatrogenic brachial plexus injuries, non-traumatic plexopathies, and incomplete medical records were excluded. After applying the predefined inclusion and exclusion criteria, 75 adult patients with traumatic brachial plexus injuries constituted the final study cohort (Figure 1).

Data collection

Data were retrospectively retrieved from hospital records using a structured data extraction pro forma. Demographic characteristics, injury-related variables, clinical classification, associated injuries, and management details were recorded (Table 1).

**Table 1:** Variables recorded in patients with brachial plexus injury The data represent original findings from the AIIMS Bathinda cohort. Institutional Ethics Committee approval was obtained (IEC/AIIMS/BTI/2025/09/07).

Parameters recorded
Age
Sex
Address / Region
Side involved
Mode and mechanism of injury
Aetiology (road traffic accident, fall, obstetric, others)
Level of brachial plexus involvement
Type of injury (complete / incomplete)
Time interval between injury and presentation
Associated injuries
Management (conservative / surgical)
Type of surgical procedure performed

The diagnosis and level of brachial plexus involvement were determined primarily through detailed clinical examination, including assessment of motor function using the Medical Research Council (MRC) grading system [8], evaluation of sensory deficits, and analysis of muscle involvement corresponding to specific root levels. Radiological investigations, including magnetic resonance imaging (MRI), were used to identify root avulsions, pseudomeningoceles, and postganglionic injuries. Electrodiagnostic studies, including nerve conduction studies and electromyography, were used to support clinical localization.

**Figure 2:**
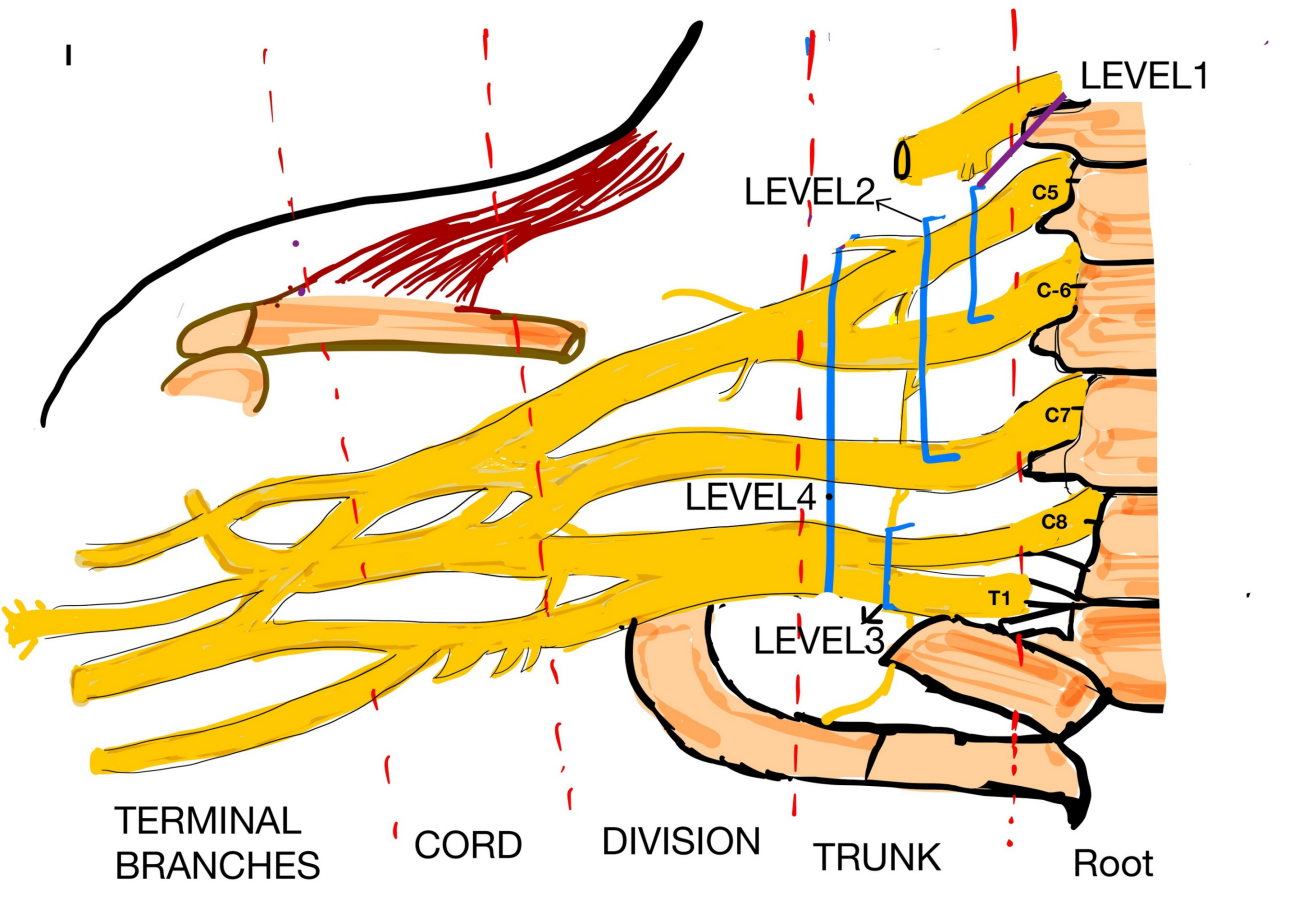
Schematic representation of the brachial plexus illustrating anatomical levels and corresponding injury patterns Level 1 represents upper plexus injury involving C5–C6 roots. Level 2 represents extended upper plexus injury involving C5–C7 roots. Level 3 represents lower plexus injury involving C8–T1 roots. Level 4 represents complete plexus injury involving C5–T1 roots. The diagram demonstrates the root, trunk, division, cord, and terminal branch components to aid visualization of anatomical distribution.

For analytical purposes, complete plexus injury was defined as involvement of the C5-T1 roots, typically presenting clinically as a flail limb. Upper plexus injury was defined as involvement of C5-C6 or C5-C7 roots, whereas lower plexus injury referred to involvement of C8-T1 roots (Figure 2).

The time from injury to presentation was categorized into five predefined intervals: <3 months, 3-6 months, 6-9 months, 9-12 months, and >12 months.

Statistical analysis

Statistical analysis was performed using IBM Corp. Released 2023. IBM SPSS Statistics for Windows, Version 29. Armonk, NY: IBM Corp. Given the descriptive nature of the study, categorical variables were summarized using frequencies and percentages. Continuous variables, such as age, were expressed as mean and range. No inferential statistical testing was performed. Graphical representations were generated to illustrate the distribution of epidemiological variables.

Ethical considerations

The study protocol was reviewed and approved by the Institutional Ethics Committee (IEC Approval No. IEC/AIIMS/BTI/2025/09/07). Patient confidentiality was maintained throughout the study, and all data were anonymized prior to the analysis.

## Results

Demographic characteristics

**Figure 3:**
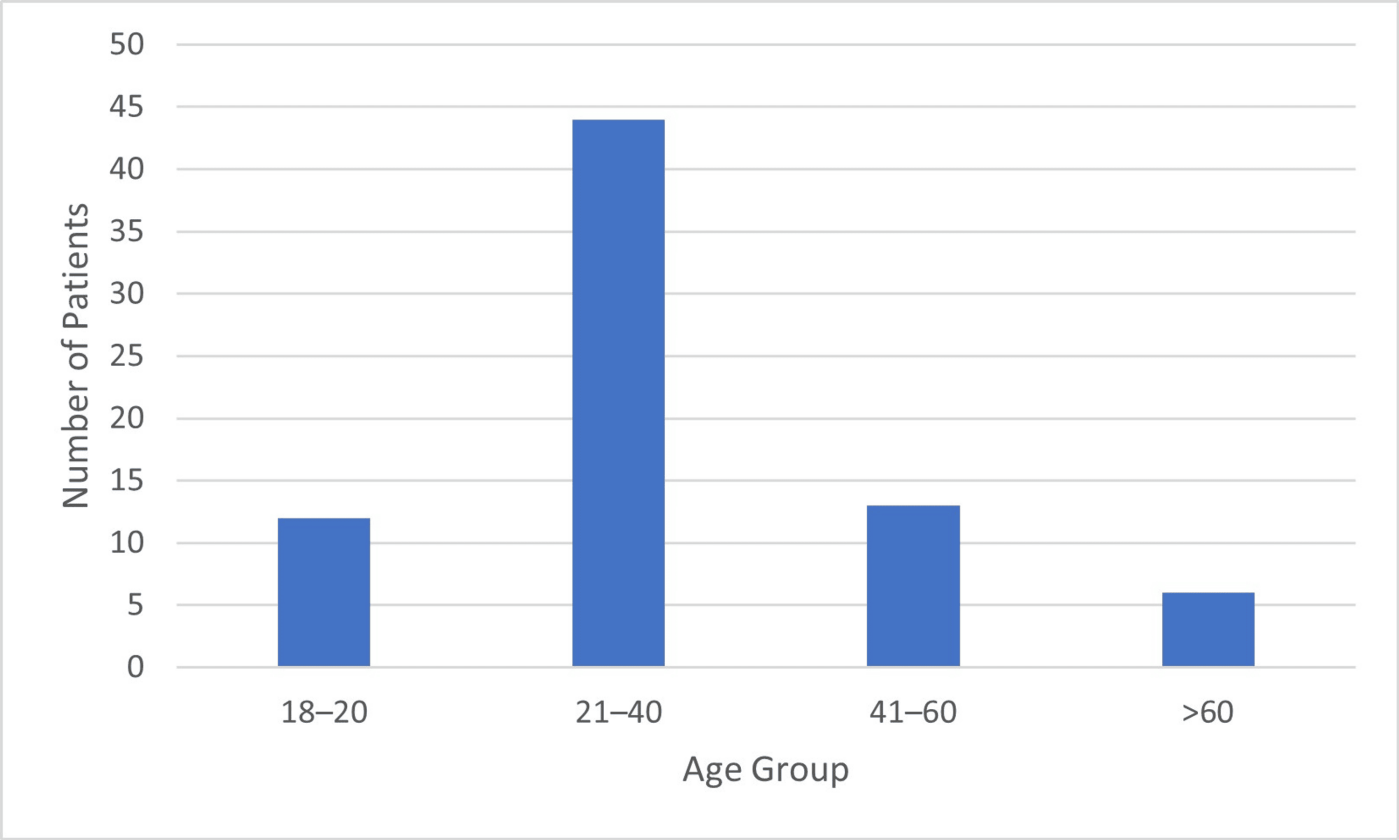
Age-wise distribution of patients with brachial plexus injury in the study population

Seventy-five adult patients with traumatic brachial plexus injury were included in the final analysis. Age ranged from 18 to 68 years, with a mean age of 29.1 years. The majority of patients belonged to the 21-40-year age group (44, 58.7%), followed by 41-60 years (13, 17.3%), 18-20 years (12, 16%), and >60 years (6, 8%) (Figure 3).

There was a marked male predominance, with 69 males (92%) and 6 females (8%).

Mechanism of injury

Road traffic accidents were the predominant mechanism, accounting for 69 cases (92%). Among these, two-wheeler accidents constituted the majority (59, 78.7%), followed by four-wheeler accidents (10, 13.3%). Non-vehicular causes included falls from height (4, 5.3%) and assault (2, 2.7%) (Table 2).

**Table 2:** Distribution of patients according to mechanism of injury Data represents original findings from the AIIMS Bathinda cohort. Institutional Ethics Committee approval was obtained (IEC/AIIMS/BTI/2025/09/07). Values are presented as numbers (percentages). Descriptive statistics were used for analysis.

Mechanism of injury	Number of patients	Percentage (%)
Two-wheeler accident	59	78.7
Four-wheeler accident	10	13.3
Fall from height	4	5.3
Assault	2	2.7
Total	75	100

Side involved

**Figure 4:**
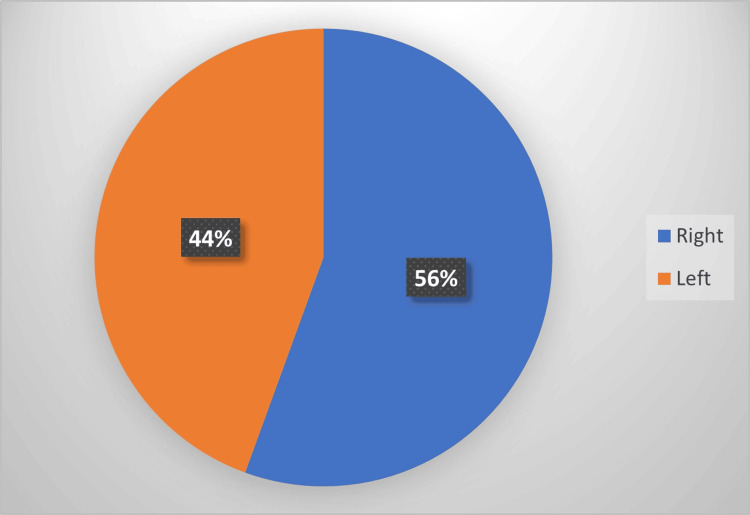
Distribution of limb laterality in adult traumatic brachial plexus injury Pie chart illustrating the proportion of right- and left-sided involvement among patients included in the study cohort.

The right upper limb was affected in 42 patients (56%), whereas the left side was involved in 33 patients (44%) (Figure 4).

Type and level of injury

Complete brachial plexus injury involving the C5-T1 roots was the most common pattern (59, 78.7%). An upper plexus injury involving C5-C6 roots was observed in 9 patients (12%), while an extended upper plexus injury (C5-C7) was seen in 6 patients (8%). A lower plexus injury (C8-T1) was identified in 1 patient (1.3%) (Table 3).

**Table 3:** Distribution of patients according to type and level of brachial plexus injury The data represent original findings from the AIIMS Bathinda cohort. Institutional Ethics Committee approval was obtained (IEC/AIIMS/BTI/2025/09/07). Values are presented as numbers (percentages). Descriptive statistics were used for analysis.

Type and level of injury	Number of patients	Percentage (%)
Complete plexus injury (C5–T1)	59	78.7
Upper plexus injury (C5–C6)	9	12
Upper plexus injury (C5, C6, C7)	6	8
Lower plexus injury (C8–T1)	1	1.3
Total	75	100

Time to presentation

**Figure 5:**
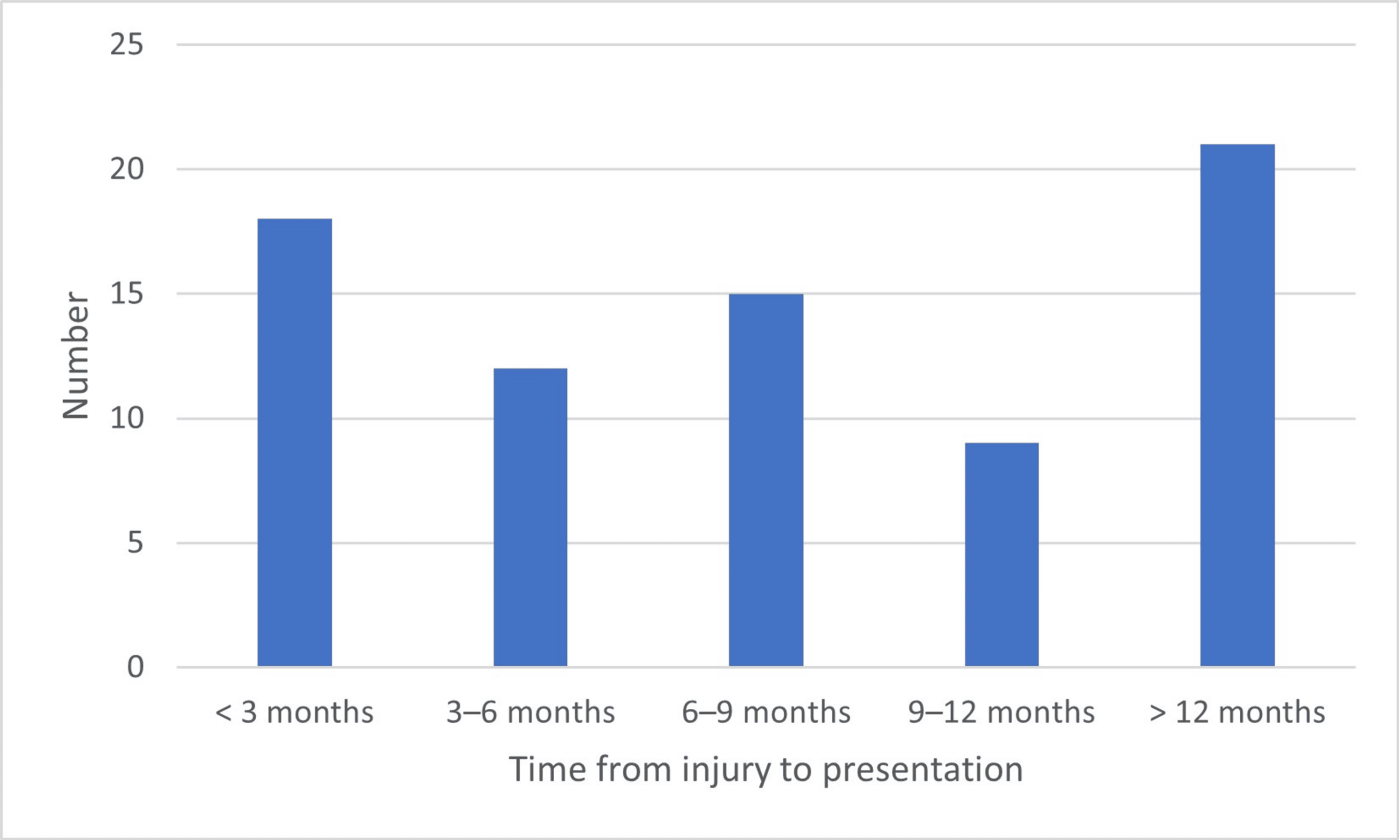
Distribution of patients according to time interval between injury and presentation (n = 75)

A substantial delay in presentation was observed. Eighteen patients (24%) presented within three months of injury, and 12 (16%) presented between three and six months. Fifteen patients (20%) presented between six and nine months, nine (12%) between nine and twelve months, and 21 (28%) presented more than twelve months after injury. Overall, 45 patients (60%) presented beyond six months (Figure 5).

Associated injuries

Associated injuries were present in 42 patients (56%), whereas 33 (44%) had isolated brachial plexus injuries. Shoulder girdle injuries were the most common (21, 28%), followed by upper limb fractures (17, 22.7%), rib fractures (9, 12%), lower limb fractures (8, 10.6%), polytrauma (5, 6.6%), facial injuries (4, 5.3%), and spinal injuries (4, 5.3%) (Table 4).

**Table 4:** Distribution of associated fractures and injuries in patients with brachial plexus injury Data represent original findings from the AIIMS Bathinda cohort. Institutional Ethics Committee approval was obtained (IEC/AIIMS/BTI/2025/09/07). Values are presented as numbers (percentages). Descriptive statistics were used for analysis. Some patients sustained more than one associated injury.

Associated fracture / injury	Number of patients	Percentage (%)
Shoulder girdle	21	28
Upper limb	17	22.7
Lower limb	8	10.6
Rib	9	12
Facial	4	5.3
Polytrauma	5	6.6
Spine	4	5.3

Management profile

At the time of analysis, 51 patients (68%) had undergone surgical intervention, whereas 24 (32%) had not undergone operative treatment.

A total of 54 nerve transfer procedures were performed, either alone or in combination. The most commonly performed procedure was spinal accessory nerve (SAN) to suprascapular nerve (SSN) transfer (19, 37.3% of operated cases), followed by triceps motor branch to axillary nerve transfer (14, 27.4%), intercostal nerve (ICN) to musculocutaneous nerve (MCN) transfer (11, 21.6%), trapezius transfer (11, 21.6%), and Oberlin transfer (10, 19.6%). Secondary reconstructive procedures included wrist arthrodesis (4, 7.8%) and free functional gracilis muscle transfer (2, 3.9%). Some patients underwent more than one procedure (Table 5).

**Table 5:** Distribution of surgical procedures performed on patients with brachial plexus injury (n = 51*) Data represent original findings from the AIIMS Bathinda cohort. Institutional Ethics Committee approval was obtained (IEC/AIIMS/BTI/2025/09/07). Values are presented as numbers (percentages). Descriptive statistics were used for analysis. SAN: spinal accessory nerve; SSN: suprascapular nerve; ICN: intercostal nerve; MCN: musculocutaneous nerve. *Some patients underwent more than one surgical procedure.

Surgical procedure	Number of patients	Percentage (%)
Spinal accessory nerve (SAN) to suprascapular nerve (SSN) transfer	19	37.3
Intercostal nerve (ICN) to musculocutaneous nerve (MCN) transfer	11	21.6
Trapezius transfer	11	21.6
Oberlin transfer	10	19.6
Triceps motor branch → axillary nerve transfer	14	27.4
Wrist arthrodesis	4	7.8
Free functional Gracilis muscle transfer	2	3.9

## Discussion

Adult traumatic brachial plexus injuries (BPI) remain among the most complex peripheral nerve injuries, resulting in profound upper limb dysfunction and long-term socioeconomic consequences. The present study provides region-specific data from a tertiary care center in semi-urban Punjab and highlights several key findings: marked male predominance, overwhelming association with high-velocity road traffic accidents, a high proportion of complete plexus injuries, substantial delay in presentation, and a significant reconstructive burden at the tertiary level.

In our cohort, the mean age was 29.1 years, and more than 90% of patients were male. This demographic distribution is consistent with both Indian and international literature, which consistently demonstrates that traumatic BPI predominantly affects young adult males exposed to high-energy vehicular trauma [2-7]. The predominance of two-wheeler accidents (78.7%) in our study mirrors earlier Indian data [2] as well as large international cohorts [5,7], reinforcing the strong association between motorcycle-related trauma and severe plexus injuries. Population-based data from England and Wales have similarly demonstrated a persistent burden of traumatic BPI over several decades, particularly in young males involved in high-velocity accidents [6].

A notable finding in our series was the high proportion of complete C5-T1 injuries (78.7%). This percentage appears higher than that reported in some population-based datasets [6,9], likely reflecting referral bias to a tertiary reconstructive center and the severity of trauma patterns in our region. The mechanism of injury plays a crucial role in determining anatomical involvement. During high-speed motorcycle crashes, forceful depression of the shoulder combined with contralateral cervical flexion generates intense longitudinal traction across the brachial plexus. This traction mechanism can produce multi-root avulsion or complete plexus palsy, particularly when the energy transfer is substantial [5,7,10]. The predominance of complete injuries in our cohort likely reflects this high-energy traction mechanism coupled with the selective referral of more severe cases to specialized centers.

The reconstructive implications of complete plexus involvement are substantial. Such injuries often require multiple nerve transfers or secondary reconstructive procedures, increasing operative complexity and rehabilitation demands. Contemporary trends in brachial plexus surgery emphasize nerve transfers as primary strategies for the restoration of shoulder and elbow function [3,5], and the high operative rate observed in our series reflects this evolving reconstructive paradigm. Increasing procedural volumes and healthcare expenditures associated with brachial plexus reconstruction have been documented in national datasets [11,12], further underscoring the healthcare resource implications of these injuries.

Delayed presentation remains a recurring challenge in developing healthcare systems. In our study, 60% of patients presented beyond six months after injury, and nearly one-third presented after twelve months. Although early nerve reconstruction within 3-6 months is considered ideal for maximizing reinnervation potential [1], delayed presentation does not necessarily preclude surgical intervention. Secondary reconstructive options, including tendon transfers and free functional muscle transfer, remain viable alternatives in selected patients. However, delayed referral may necessitate a shift from primary nerve reconstruction to more complex salvage procedures, potentially affecting ultimate functional recovery.

The high proportion of delayed presentations in our cohort likely reflects multifactorial influences, including initial management at peripheral centers, delayed recognition of the severity of plexus injury, socioeconomic constraints, polytrauma-related priorities, and limited awareness regarding the optimal timing of specialist referral. Similar referral delays have been documented in other developing regions [13]. Trauma registry data also suggest that brachial plexus injuries are frequently identified in the context of multisystem trauma, where immediate life-saving priorities may overshadow early neurological referral [14]. Strengthening referral pathways and increasing awareness among primary care and trauma providers may improve early surgical consultation and expand reconstructive options.

Nearly one-third of patients in our cohort had not undergone surgical intervention at the time of analysis. This reflects the complexity of decision-making in adult traumatic BPI, where management is individualized based on timing, injury severity, residual motor function, patient expectations, and rehabilitation capacity. In late-presenting cases, anticipated functional gains and rehabilitation burden must be balanced against surgical morbidity. This individualized approach highlights that reconstructive workload extends beyond operative volume and includes long-term multidisciplinary care.

Associated injuries were common, particularly involving the shoulder girdle and upper limb, reflecting the high-energy trauma responsible for plexus disruption. Concomitant fractures, dislocations, and polytrauma may delay neurological evaluation and influence surgical timing. These findings emphasize the importance of multidisciplinary coordination between trauma, orthopedic, and reconstructive teams.

By documenting both injury severity distribution and operative intervention patterns within the same cohort, this study provides a comprehensive overview of the epidemiological and reconstructive landscape of adult traumatic BPI in a semi-urban tertiary center. Region-specific data such as these are essential for planning resource allocation, optimizing referral networks, and designing preventive road safety strategies.

Study limitations

This study has several limitations. Its retrospective design limits control over data completeness and uniformity of documentation. The single-center setting may reduce generalizability and introduce referral bias, potentially contributing to the higher proportion of severe and complete injuries observed. The relatively small sample size restricts robust subgroup analyses and broader epidemiological inference. Functional outcomes were not systematically assessed using standardized long-term evaluation tools, and follow-up data were incomplete for some patients. Additionally, a formal correlation between clinical, radiological, and intraoperative findings could not be consistently established because of non-uniform documentation inherent to retrospective record reviews.

Future directions

Prospective multicenter studies incorporating standardized functional outcome measures and long-term follow-up are needed to better define prognostic indicators and evaluate the effectiveness of reconstructive strategies. Public health initiatives focused on motorcycle safety, early referral systems, and trauma network strengthening may help reduce the burden of severe brachial plexus injuries in semi-urban populations.

## Conclusions

Adult traumatic brachial plexus injury in our setting represents a predominantly high-energy trauma pattern affecting young males and imposing a substantial reconstructive burden on tertiary care services. The high proportion of complete plexus injuries and the frequent delay in presentation highlight the challenges of timely referral and optimal surgical planning in semi-urban healthcare systems. Strengthening early referral pathways, improving trauma awareness, and enhancing multidisciplinary coordination may help optimize functional outcomes. Prospective multicenter studies incorporating standardized outcome assessment are needed to better define regional epidemiological trends and refine reconstructive strategies in similar healthcare settings.
